# Propofol infusion syndrome resuscitation with extracorporeal life support: a case report and review of the literature

**DOI:** 10.1186/2110-5820-3-32

**Published:** 2013-09-23

**Authors:** Michael Mayette, Jeremy Gonda, Joe L Hsu, Frederick G Mihm

**Affiliations:** 1 Divisions of Pulmonary and Critical Care Medicine, Critical Care Medicine and Anesthesia, Stanford University School of Medicine, Stanford, CA 94305, USA; 2 Department of Anesthesiology, Pain and Perioperative Medicine, Stanford Hospital & Clinics, 300 Pasteur Drive, Stanford, CA 94305, USA

**Keywords:** Propofol infusion syndrome, Cardiogenic shock, Extracorporeal membrane oxygenation

## Abstract

We report a case of propofol infusion syndrome (PRIS) in a young female treated for status epilepticus. In this case, PRIS rapidly evolved to full cardiovascular collapse despite aggressive supportive care in the intensive care unit, as well as prompt discontinuation of the offending agent. She progressed to refractory cardiac arrest requiring emergent initiation of venoarterial extracorporeal membrane oxygenation (ECMO) during cardiopulmonary resuscitation (CPR). She regained a perfusing rhythm after prolonged (>8 hours) asystole, was weaned off ECMO and eventually all life support, and was discharged to home. We also present a review of the available literature on the use of ECMO for PRIS.

## Introduction

Propofol infusion, frequently used in the intensive care setting for sedation or for refractory status epilepticus, has been associated with a rare, but grave complication: propofol infusion syndrome (PRIS). We describe a case of particularly severe PRIS that was successfully resuscitated with extracorporeal membrane oxygenation (ECMO), despite prolonged evidence of cardiopulmonary arrest (asystole). This case highlights the potential severity of this syndrome, the benefits of aggressive treatment, which may include extracorporeal circulation, but also the debilitating complications associated with ECMO.

### Case report

The patient is a 20-year-old Caucasian female with a past medical history significant only for anemia who presented to a referring center’s emergency department (ED) with persistent spinal headache following a normal spontaneous vaginal delivery managed with epidural analgesia. She underwent a blood patch on postpartum day 1, which was repeated on postpartum day 3 without relief of her headache. She returned to the ED postpartum day 5 for continued headaches and was admitted for bed rest and caffeine therapy. On the night of admission, the patient started seizing (tonic-clonic) and became apneic requiring intubation and intensive care unit (ICU) admission. The seizures abated before administration of lorazepam, but she had a second seizure and was placed on a midazolam and propofol infusion for sedation and seizure control. In consultation with neurology, she was started on phenytoin and valproic acid. Ceftriaxone and vancomycin were started empirically for meningitis; both were stopped early, because cultures remained negative. A magnetic resonance imaging (MRI) study of her lumbar spine showed a cerebral spinal fluid (CSF) leak with evidence of CSF anterior and posterior to the thecal sac from the upper lumbar spine extending down to the sacrum. A brain MRI demonstrated signs of intracranial hypotension without evidence of hemorrhage. Magnetic resonance venography (MRV) of the brain was negative for venous thrombosis. Neurosurgery was consulted for possible surgical repair of the CSF leak but determined that the leak could be managed medically with bed rest, Trendelenburg positioning, and a larger blood patch. During the next 12–24 hours, the patient continued to present seizures as demonstrated by spot electroencephalography (EEG). Her propofol infusion was increased to 150 μg/kg/min (9 mg/kg/h) in an attempt to achieve epileptic burst suppression. The following day, the patient rapidly developed fluid resistant shock, a severe metabolic acidosis, acute oliguric kidney failure, an elevated creatine kinase, and transaminases. Propofol was discontinued given the concern for possible PRIS; she was resuscitated with crystalloid and started on an epinephrine drip titrated to 150 ng/kg/min to maintain a mean arterial pressure >65 mmHg. She was then emergently transferred to an academic tertiary referral center for a higher level of care.

**Figure 1:**
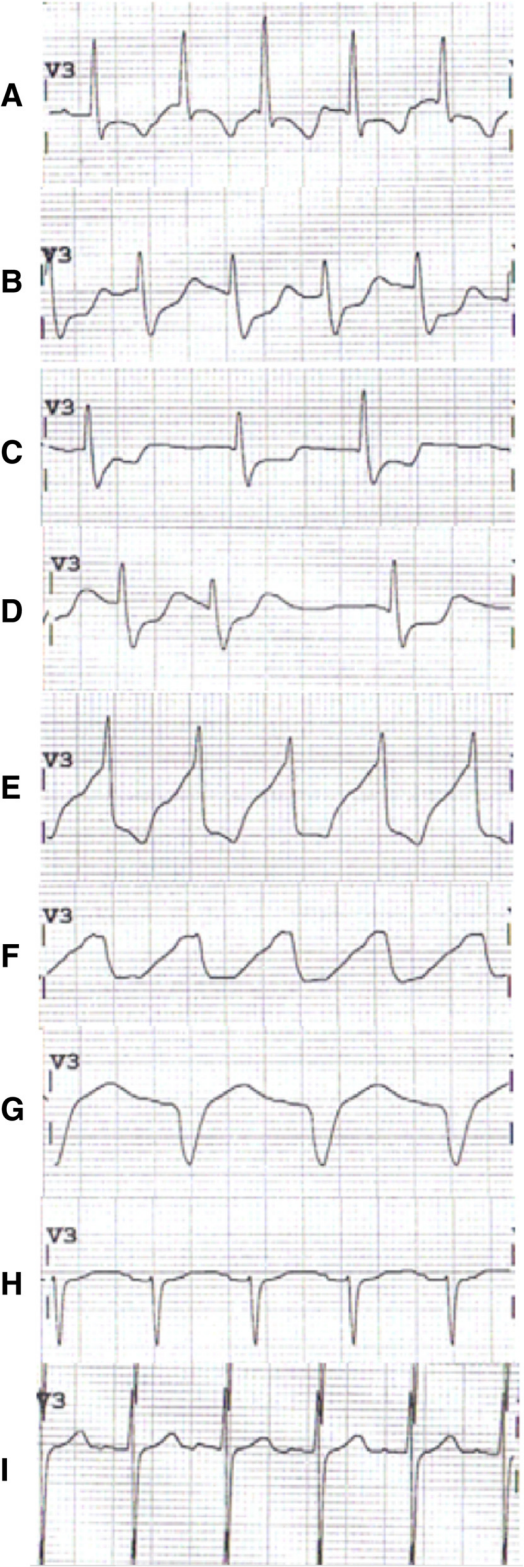
**Sequential appearance of ECG over time in V3 lead. A** On admission to our center. Sinus tachycardia with T-wave inversion (TWI). **B** Admission + 6 hours. Sinus tachycardia with ST depression and prolonged QTc. **C** Admission + 9 hours. Junctional rhythm with ongoing ST depression/TWI. **D** Admission + 12 hours. Same as **C**. **E** Admission + 18 hours. Polymorphic wide complex tachycardia with QRS slurring. **F** Admission + 24 hours (ECMO starts minutes after this ECG). Monomorphic ventricular tachycardia with sinusoidal appearance. **G** Admission + 3 days, on ECMO. Nonspecific intraventricular conduction delay. **H** Admission + 8 days, after decannulation from ECMO circuit. Sinus tachycardia with now narrowed QRS complex and T-wave flattening. **I** Admission + 60 days. Sinus tachycardia.

Significant labs at time of transfer included: serum bicarbonate 10 mmol/L, lactate 11 mg/dL, a creatine kinase (CK) of 341,000 U/L, arterial blood gas (ABG) 7.37/20/147/11/Base deficit 14 (on pressure control ventilation (PCV), peak inspiratory pressure of 15 cmH_2_O, positive end expiratory pressure of 5 cmH_2_O, FiO_2_ 50%), white blood cells count (WBC) 32 × 10^9^/L, AST 3827 U/L, ALT 789 U/L, alkaline phosphatase 193 U/L, total bilirubin 0.6 mg/dL, INR 1.4. Her initial ECG was concerning for a new onset right bundle branch block with left anterior fascicular block (Figure 1A and B).

During the first 8 hours after transfer, her severe acidosis and acute liver failure remained stable and her lactate level improved, but she became oliguric with mild hyperkalemia (5.1 mmol/L) and hypocalcemia (ionized 0.76 mmol/L). Troponins peaked at 15.5 ng/mL (normal < 0.3 ng/mL) on post-transfer day 1. She was placed on continuous renal replacement therapy (CRRT). Cardiology, neurology, and cardiothoracic surgery were all consulted with early discussions of possibly placing the patient on extracorporeal membrane oxygenation (ECMO) or other mechanical cardiac support as a rescue therapy should she worsen. Propofol having been discontinued before transfer to our facility, she was not placed on any other continuous sedation regimen. She started to awaken and move all extremities upon arrival with intermittent following of commands, and only required intermittent boluses of opioids for adequate sedation. A continuous EEG did not show any ongoing seizure activity. Approximately 8 hours after transfer, the patient suffered an episode of unstable ventricular tachycardia (V-tach) requiring three defibrillation attempts (200 Joules biphasic) without CPR (maintained pulse) resulting in return of a sinus tachycardia with a persistent right bundle branch block (RBBB) and septal lead ST-segment depression (Figure 1C). She was loaded with amiodarone and given aggressive calcium/magnesium replacements. Over the subsequent 6 hours, she continued to have a very irritable myocardium/conduction system resulting in atypical intraventricular conduction delays (IVCDs), progressively widening QRS, atrial flutter/fibrillation, and an accelerated junctional escape rhythm (Figure 1D,E,F).

At approximately 16:30 that evening, 18 hours after transfer, the patient suffered another V-tach episode, this time pulseless, requiring 10 minutes of CPR, 6 (1 mg) doses of epinephrine, defibrillations, and 100 mg of IV lidocaine with return of spontaneous circulation. She remained in an accelerated junctional rhythm/V-tach with a perfusing blood pressure (101/64 mmHg, heart rate 135 beats per minute). Because of her continued circulatory and electrophysiologic instability, the decision was made to place a temporary ventricular assist device (VAD; Impella, Abiomed®) under fluoroscopic guidance in the cardiac catheterization laboratory. As she was being prepared to transfer out of the ICU, she went into ventricular fibrillation (V-fib). Again, resuscitation attempts were continued with continuous CPR, epinephrine, and ventilation. The decision was made to place her emergently onto ECMO while undergoing active advanced cardiac life support (ACLS) resuscitation. She was cannulated at the bedside using a percutaneously placed right internal jugular venous catheter and a left femoral arterial catheter for venous-arterial (V-A) ECMO. During cannulation, the patient’s rhythm degraded into a fine V-fib and she became asystolic. Cardiopulmonary resuscitation was continued and after being loaded with 10,000 units of heparin, the patient was placed on the ECMO circuit while still asystolic and remained with minimal cardiac motion as seen on bedside echocardiography. The time from the start of her V-fib arrest to ECMO initiation was 53 minutes with minimal interruptions in CPR and ongoing boluses of epinephrine, vasopressin, bicarbonate, and calcium chloride. Postcardiac arrest therapeutic hypothermia protocol to 32°C for 24 hours was initiated via the ECMO circuit for neurologic preservation.

**Figure 2:**
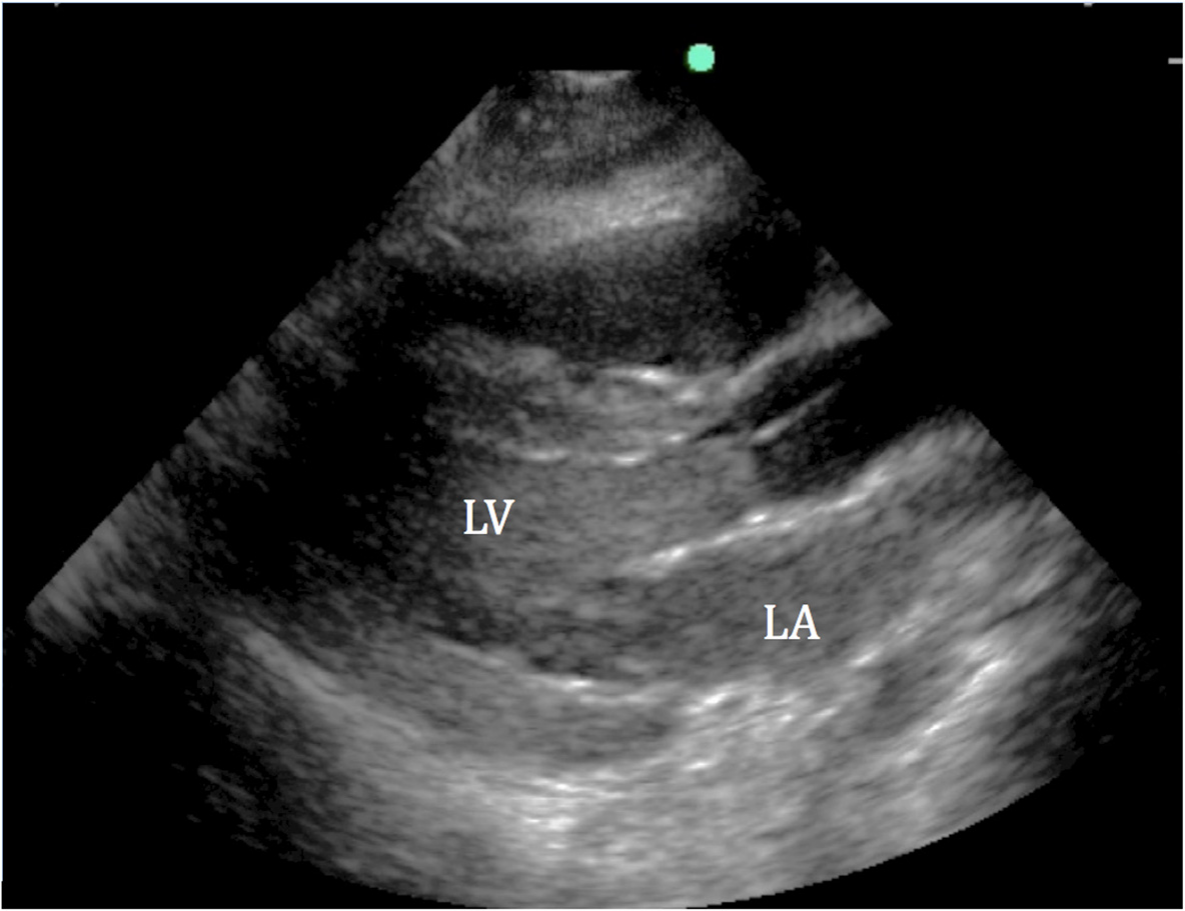
**Transthoracic parasternal long axis view performed at bedside during asystole while on ECMO circuit.** Note the “smoky appearance” in the left atrium (LA) and left ventricle (LV) concerning for developing thrombus.

At this time, her labs returned and she was found to have serum potassium of 8.8 mmol/L (nonhemolyzed) despite ongoing CRRT. Her CK level peaked at 655,200 U/L. She was given additional calcium chloride, bicarbonate, insulin, glucose, sodium polystyrene sulfonate (Kayexalate®), and the CRRT (in continuous veno-venous hemodiafiltration mode, or CVVHDF) flows were increased. Despite dialyzing with a potassium bath of zero mEq/L, with blood flow rate of 300 mL/min, pre- and post-filter flows of 800 mL/h and 400 mL/h respectively and dialysate flow of 1,200 mL/h, along with ongoing medical management for the hyperkalemia, her potassium remained >8 mEq/L for the next 6 hours. Because of a lack of cardiac activity activated clotting times (ACT) were attempted to be maintained at >200 seconds. Frequent assessments of cardiac activity using bedside transthoracic echocardiography (TTE) confirmed prolonged and persistent asystole (see Additional file 1). Concern arose for thrombus formation given prolonged cardiac standstill and as the patient was requiring escalating doses of heparin to achieve the target ACT, anticoagulation was switched to argatroban. A bedside TTE at the time showed a “smoke-like signal” in the left ventricle (LV) concerning for LV thrombus formation (Figure 2).

In addition to the ongoing efforts to decrease her serum potassium, we also attempted unsuccessfully to chemically pace her heart using isoproterenol, epinephrine, and dopamine infusions. Transvenous pacing also was employed without successful electrical capture of the heart. Because of the continued inability to lower the potassium with CRRT the patient was transitioned to intermittent hemodialysis (iHD). During iHD, her potassium finally responded and dropped to 4.5 mEq/L within 1 hour into the dialysis treatment and she regained a paced rhythm. She regained a rapidly improving pulse pressure on arterial line tracing as well as evidence of aortic valve opening on TTE.

After being on ECMO for 5 days, the patient demonstrated a dramatic recovery of cardiac function by TTE with an LVEF 35-40% and normal RV function, facilitating weaning, and removal from the ECMO circuit [1,2]. She was successfully decannulated from the ECMO circuit to low-dose vasopressors that were rapidly weaned by day 7. Perfusion to her lower extremities (LE) was compromised during ECMO with hypothermia and high-dose pressors, despite the early use of a reperfusion catheter distal to cannulation site (forward limb). Because of concerns for compartment syndrome in left LE (ECMO arterial cannulation site), she underwent fasciotomies. Unfortunately, she eventually required a left below-the-knee amputation (BKA) and a left ileofemoral bypass surgery. She also underwent a brain MRI which showed left frontal and right occipital ischemic strokes, concerning for embolic events or cerebral hypoperfusion.

Despite her complicated ICU course, she gradually awoke with significant residual weakness and paralysis in the left upper extremity. She eventually recovered normal renal function after 1 month of dialysis requirement (creatinine 0.5 mg/dL on follow-up), as well as normal liver function. Her repeat ECGs showed a sinus rhythm with minimal lateral T-wave abnormalities (Figure 1H and I). Outpatient echocardiogram showed normal LV size, wall thickness and contractility, with estimated EF 60%. She had short-term memory deficits and some depression/anxiety from the events but was cognitively intact. During her prolonged hospitalization, she also suffered from *Clostridium difficile* colitis and developed diffuse calcified lesions in her lungs that were felt to be secondary to amiodarone toxicity. She was transferred from our hospital to rehabilitation after 84 days of hospitalization.

## Discussion

Propofol (2,6-diisopropylphenol) is a commonly used sedative and hypnotic drug in the ICU, allowing easily titratable continuous sedation [3-5]. It has been FDA approved since 1993 for sedation induction and maintenance in anesthesia and intensive care. Its short half-life allows for frequent neurological assessment. Because of the strong antiepileptic and neuro-protective properties of the drug, it is frequently used for treatment of refractory status epilepticus [6-8]. Neuroprotection is thought to be due to inactivation of gamma-aminobutyric acid (GABA) receptors, blockade of excitatory neurotransmitters, reduction in cerebral oxygen consumption and intracranial pressure [9].

Although propofol has appealing properties as a first line drug for sedation, its clinical use has been limited because of adverse effects. Significant hypotension, especially in the hemodynamically unstable patient from both peripheral vasodilation and cardiac depression, hinders its use. Hypertriglyceridemia and associated complications also are observed in some patients. The clinical effect and occurrence of side effects of propofol are generally dose- and time-dependent [10].

A rare complication, propofol infusion syndrome (PRIS), was first described in 1992 [11], and the name PRIS was first used in 1998 [12]. Initially described in children [11-13] and in traumatic brain injury [14], it has been increasingly reported in critically ill adults [15,16], including during treatment for refractory status epilepticus. Propofol infusion syndrome typically presents as severe rhabdomyolysis, acute kidney injury, hyperkalemia, metabolic acidosis, and hepatomegaly. Myocardial injury may occur in severe forms, presenting with various ECG changes, including Brugada-like pattern and coved-type ST-segment elevations, severe arrhythmias, and cardiovascular collapse [17-19]. Occurrence of the syndrome, as well as its severity, appears to be dose-dependent, most cases occurring in patients who received a propofol dose in excess of 5 mg/kg/hr (80 μg/kg/min) for at least 48 hours [20]. However, as in our case, the syndrome has been described with short-term, high doses [21] and long-term, small doses [22]. Additional well-recognized risk factors for its development include the coadministration of catecholamines or corticosteroids [20,23].

As this constellation of signs and symptoms remains nonspecific and in the absence of a definitive diagnostic test, PRIS is always a presumed diagnosis and a differential diagnosis must always be kept in mind. This includes septic shock with end-organ hypoperfusion, trauma with crush injuries, ongoing seizures, toxic rhabdomyolysis (cocaine, amphetamines), severe alcohol withdrawal/delirium tremens with cardiovascular collapse, tetanus, or severe hypophosphatemia.

The pathophysiology of PRIS is complex. Propofol infusion syndrome has a higher incidence in critically ill patients compared with healthy patients receiving propofol for routine anesthesia. Hence, critical illness, most notably neurologic injury, might act as a priming factor for propofol (with or without catecholamines and corticosteroids) to trigger PRIS [20]. The end-organ damage may result from an imbalance between energy demand and oxygen utilization. Propofol, by increasing malonylcarnitine, impairs uptake and mitochondrial oxidation of long-chain free fatty acids (FFAs) in myocardial and skeletal muscle, resulting in muscle necrosis [24]. Critically ill patients are particularly dependent on FFAs for energy production, and the generation of FFAs from peripheral lipolysis is catecholamine-driven. Propofol also appears to directly inhibit the respiratory electron transport chain in cardiac mitochondria, inhibiting utilization of freely diffusing medium- and short-chain FFAs [25]. The role of catecholamines in the syndrome may be related to direct muscle damage (contraction band necrosis), as seen in other high-catecholamine states like pheochromocytoma [26] and stress-induced (Tako-Tsubo) cardiomyopathy [27,28]. Neurologic injury, frequently present in patients suffering from PRIS, also is known to produce a high-catecholamine state with associated muscle damage [29]. Finally, a genetic predisposition of inappropriate fatty acid oxidation (inborn error) has been hypothesized but remains uncertain [24,30].

Treatment of PRIS first and foremost relies on rapid recognition of the syndrome and removal of the offending agent. Otherwise, the treatment is mainly supportive by the use of inotropic and vasopressive agents, aggressive electrolyte control, hemodialysis, or hemofiltration. As mentioned above, severe forms of PRIS with cardiac injuries and arrhythmias have been reported, with various arrhythmias including asystole [17], as in our case. For those severe forms of cardiovascular collapse, mechanical circulatory support, using VADs or ECMO has been reported. Extra corporeal membrane oxygenation, especially VA-ECMO as used in this case, can be deployed to temporarily support end-organ blood flow and oxygen delivery using either central or peripheral cannulation [31]. This remains a high-risk procedure, frequently used *in extremis* when other treatment modalities have failed, or when the patient is too unstable to be transferred to the catheterization laboratory, as in our case. The risk of complications (mechanical complications of the circuit, hemorrhage from anticoagulation and consumptive coagulopathy, vascular trauma induced by large-bore catheters, local thrombosis with limb ischemia and systemic emboli, sepsis) remains significant despite advances in the technique [32]. We found three case reports of the use of ECMO for severe PRIS, two in pediatric populations and one in a young adult [33-35]. To our knowledge, this is the first case of such prolonged documented asystole with survival, despite significant complications.

## Review and conclusions

Overall reported mortality for PRIS, usually associated with the cardiac injury, is as high as 64% [17]. The simultaneous presence of multiple characteristics of the syndrome is associated with increased mortality [36], highlighting the need for education about PRIS and early recognition and treatment. Repeated case reports of this devastating complication of a common ICU drug calls for increased caution and vigilance when using propofol, especially in patients requiring increasing doses of vasopressors or in cardiac failure as advised by the American College of Critical Care Medicine [3]. Many centers have modified protocols of treatment, including refractory status epilepticus, suggesting propofol should be moved to second or third-line choice when alternatives exist. When used, small doses for short periods of time should be the norm. Frequent monitoring of CK and lactate levels, as well as clinical awareness needs to be encouraged. For severe cases with cardiovascular collapse, strong consideration should be given to early transfer to medical centers with capacities for advanced mechanical circulatory support.

## Consent

Informed consent was obtained from the patient for the publication of this report and any accompanying images.

## Abbreviations

PRIS: Propofol infusion syndrome; ECMO: Extracorporeal membrane oxygenation; CPR: Cardiopulmonary resuscitation; ED: Emergency department; ICU: Intensive care unit; MRI: Magnetic resonance imaging; CSF: Cerebral spinal fluid; MRV: Magnetic resonance venography; EEG: Electroencephalogram; ABG: Arterial blood gas; PCV: Pressure-controlled ventilation; PIP: Peek inspiratory pressure; PEEP: Positive end-expiratory pressure; FiO2: Fraction of inspired oxygen; WBC: White blood cell; AST: Aspartate aminotransferase; ALT: Alanine transaminase; INR: Internation normalized ratio; ECG: Electrocardiogram; CRRT: Continuous renal replacement therapy; RBBB: Right bundle branch block; IVCD: Intraventricular conduction delay; VAD: Ventricular-assist device; ACLS: Advanced cardiac life support; V-A: Veno-arterial; CK: Creatine kinase; CVVHDF: Continuous veno-venous hemodiafiltration; ACT: Activated clotting time; TTE: Transthoracic echocardiogram; LV: Left ventricle; iHD: Intermittent hemodialysis; LVEF: Left ventricular ejection fraction; RV: Right ventricle; LE: Lower extremity; BKA: Below-knee amputation; FDA: Food and drug administration; GABA: Gamma-aminobutyric acid; FFA: Free fatty acids.

## Competing interests

The authors declare that they have no competing interests.

## Authors’ contributions

MM and JG wrote the initial manuscript, which was reviewed and edited by JLH and FGM. All authors read and approved the final manuscript and participated in the bedside care of the described patient. 

## Supplementary Material

Additional file 1 Parasternal long axis transthoracic echo video clip observed during 8 hr period of asystole while on ECMO support. Click here for file

## References

[B1] Firstenberg MS Orsinelli DA ECMO and ECHO: the evolving role of quantitative echocardiography in the management of patients requiring extracorporeal membrane oxygenation J Am Soc Echocardiogr 2012 3 641 643 10.1016/j.echo.2012.04.005 22625213

[B2] Platts DG Sedgwick JF Burstow DJ Mullany DV Fraser JF The role of echocardiography in the management of patients supported by extracorporeal membrane oxygenation J Am Soc Echocardiogr 2012 3 131 141 10.1016/j.echo.2011.11.009 22169046

[B3] Jacobi J Fraser GL Coursin DB Riker RR Fontaine D Wittbrodt ET Chalfin DB Masica MF Bjerke HS Coplin WM Crippen DW Fuchs BD Kelleher RM Marik PE Nasraway SAJ Murray MJ Peruzzi WT Lumb PD Clinical practice guidelines for the sustained use of sedatives and analgesics in the critically ill adult Crit Care Med 2002 3 119 141 10.1097/00003246-200201000-00020 11902253

[B4] Nasraway SAJ Jacobi J Murray MJ Lumb PD Sedation, analgesia, and neuromuscular blockade of the critically ill adult: Revised clinical practice guidelines for 2002 Crit Care Med 2002 3 117 118 10.1097/00003246-200201000-00019 11902252

[B5] Barr J Fraser GL Puntillo K Ely EW Gélinas C Dasta JF Davidson JE Devlin JW Kress JP Joffe AM Clinical practice guidelines for the management of pain, agitation, and delirium in adult patients in the intensive care unit Crit Care Med 2013 3 263 306 23269131 10.1097/CCM.0b013e3182783b72

[B6] Lowenstein DH The management of refractory status epilepticus: an update Epilepsia 2006 3 35 40 10.1111/j.1528-1167.2006.00658.x 17044824

[B7] Meierkord H Boon P Engelsen B Göcke K Shorvon S Tinuper P Holtkamp M EFNS guideline on the management of status epilepticus Eur J Neurol 2006 3 445 450 10.1111/j.1468-1331.2006.01397.x 16722966

[B8] Rossetti AO Reichhart MD Schaller M-D Despland P-A Bogousslavsky J Propofol treatment of refractory status epilepticus: a study of 31 episodes Epilepsia 2004 3 757 763 10.1111/j.0013-9580.2004.01904.x 15230698

[B9] Ito H Watanabe Y Isshiki A Uchino H Neuroprotective properties of propofol and midazolam, but not pentobarbital, on neuronal damage induced by forebrain ischemia, based on the GABAA receptors Acta Anaesthesiol Scand 1999 3 153 162 10.1034/j.1399-6576.1999.430206.x 10027021

[B10] Biebuyck JF Smith I White PF Nathanson M Gouldson R Propofol: an update on its clinical use Anesthesiology 1994 3 1005 1043 10.1097/00000542-199410000-00028 7943815

[B11] Parke TJ Stevens JE Rice AS Greenaway CL Bray RJ Smith PJ Waldmann CS Verghese C Metabolic acidosis and fatal myocardial failure after propofol infusion in children: five case reports BMJ 1992 3 613 616 10.1136/bmj.305.6854.613 1393073 PMC1883365

[B12] Bray RJ Propofol infusion syndrome in children Pediatr Anesth 1998 3 491 499 10.1046/j.1460-9592.1998.00282.x 9836214

[B13] Hanna JP Ramundo ML Rhabdomyolysis and hypoxia associated with prolonged propofol infusion in children Neurology 1998 3 301 303 10.1212/WNL.50.1.301 9443502

[B14] Cremer OL Moons KGM Bouman EAC Kruijswijk JE De Smet AMGA Kalkman CJ Long-term propofol infusion and cardiac failure in adult head-injured patients Lancet 2001 3 117 118 10.1016/S0140-6736(00)03547-9 11197401

[B15] Kang T Propofol infusion syndrome in critically ill patients Ann Pharmacother 2002 3 1453 1456 12196066 10.1345/aph.1A321

[B16] Eriksen J Povey HMR A case of suspected non-neurosurgical adult fatal propofol infusion syndrome Acta Anaesthesiol Scand 2006 3 117 119 10.1111/j.1399-6576.2006.00904.x 16451160

[B17] Corbett SM Montoya ID Moore FA Propofol-related infusion syndrome in intensive care patients Pharmacotherapy J Hum Pharmacol Drug Ther 2008 3 250 258 10.1592/phco.28.2.250 18225970

[B18] Tseng Y-T Hao W-R Liu J-C Hsieh M-H Propofol infusion syndrome leads to severe right heart injury and lethal arrhythmias J Exp Clin Med 2010 3 192 195 10.1016/S1878-3317(10)60030-7

[B19] Vernooy K Delhaas T Cremer OL Di Diego JM Oliva A Timmermans C Volders PG Prinzen FW Crijns HJGM Antzelevitch C Kalkman CJ Rodriguez L-M Brugada R Electrocardiographic changes predicting sudden death in propofol-related infusion syndrome Heart Rhythm 2006 3 131 137 10.1016/j.hrthm.2005.11.005 16443524 PMC1474111

[B20] Vasile B Rasulo F Candiani A Latronico N The pathophysiology of propofol infusion syndrome: a simple name for a complex syndrome Intensive Care Med 2003 3 1417 1425 10.1007/s00134-003-1905-x 12904852

[B21] Liolios A Guérit J-M Scholtes J-L Raftopoulos C Hantson P Propofol infusion syndrome associated with short-term large-dose infusion during surgical anesthesia in an adult Anesth Analg 2005 3 1804 1806 10.1213/01.ANE.0000153017.93666.BF 15920217

[B22] Merz TM Regli B Rothen H-U Felleiter P Propofol infusion syndrome—a fatal case at a low infusion rate Anesth Analg 2006 3 1050 10.1213/01.ane.0000239080.82501.c7 17000843

[B23] Casserly B O’Mahony E Timm EG Haqqie S Eisele G Urizar R Propofol infusion syndrome: an unusual cause of renal failure Am J Kidney Dis 2004 3 e98 e101 10.1053/j.ajkd.2004.08.036 15558515

[B24] Wolf A Weir P Segar P Stone J Shield J Impaired fatty acid oxidation in propofol infusion syndrome Lancet 2001 3 606 607 10.1016/S0140-6736(00)04064-2 11558490

[B25] Schenkman KA Yan S Propofol impairment of mitochondrial respiration in isolated perfused guinea pig hearts determined by reflectance spectroscopy Crit Care Med 2000 3 172 177 10.1097/00003246-200001000-00028 10667518

[B26] Bhatnagar D Carey P Pollard A Focal myositis and elevated creatine kinase levels in a patient with phaeochromocytoma Postgrad Med J 1986 3 197 198 10.1136/pgmj.62.725.197 3714605 PMC2418640

[B27] Abraham J Mudd JO Kapur N Klein K Champion HC Wittstein IS Stress cardiomyopathy after intravenous administration of catecholamines and beta-receptor agonists J Am Coll Cardiol 2009 3 1320 1325 10.1016/j.jacc.2009.02.020 19358948

[B28] Bybee KA Prasad A Stress-related cardiomyopathy syndromes Circulation 2008 3 397 409 10.1161/CIRCULATIONAHA.106.677625 18645066

[B29] Lee V Oh J Mulvagh S Wijdicks E Mechanisms in neurogenic stress cardiomyopathy after aneurysmal subarachnoid hemorrhage Neurocrit Care 2006 3 243 249 10.1385/NCC:5:3:243 17290097

[B30] Withington DE Decell MK Ayed TA A case of propofol toxicity: further evidence for a causal mechanism Pediatr Anesth 2004 3 505 508 10.1111/j.1460-9592.2004.01299.x 15153216

[B31] Marasco SF Lukas G McDonald M McMillan J Ihle B Review of ECMO (extracorporeal membrane oxygenation) support in critically ill adult patients Heart Lung Circ 2008 3 4 S41 S47 18964254 10.1016/j.hlc.2008.08.009

[B32] Pham T Combes A Rozé H Chevret S Mercat A Roch A Mourvillier B Ara-Somohano C Bastien O Zogheib E Clavel M Constan A Marie Richard J-C Brun-Buisson C Brochard L Extracorporeal membrane oxygenation for pandemic influenza A(H1N1)–induced acute respiratory distress syndrome: a cohort study and propensity-matched analysis Am J Respir Crit Care Med 2013 3 276 285 10.1164/rccm.201205-0815OC 23155145

[B33] Abrahams JMRG Acker MA Sinson GP Propofol J Neurosurg 2002 3 1160 1161 12066924

[B34] Culp KE Augoustides JG Ochroch AE Milas BL Clinical management of cardiogenic shock associated with prolonged propofol infusion Anesth Analg 2004 3 221 226 10.1213/01.ANE.0000117285.12600.C1 15281533

[B35] Guitton C Gabillet L Latour P Rigal J-C Boutoille D Al Habash O Derkinderen P Bretonniere C Villers D Propofol infusion syndrome during refractory status epilepticus in a young adult: successful ECMO resuscitation Neurocrit Care 2011 3 139 145 10.1007/s12028-010-9385-7 20499207

[B36] Fong JJ Sylvia L Ruthazer R Schumaker G Kcomt M Devlin JW Predictors of mortality in patients with suspected propofol infusion syndrome Crit Care Med 2008 3 2281 2287 2210.1097/CCM.2280b2013e318180c318181eb 10.1097/CCM.0b013e318180c1eb 18664783

